# Increased expression of αTubulin is associated with poor prognosis in patients with pancreatic cancer after surgical resection

**DOI:** 10.18632/oncotarget.10630

**Published:** 2016-07-16

**Authors:** Chao Lin, Guo-chao Zhao, Ya-dong Xu, Dan-song Wang, Da-yong Jin, Yuan Ji, Wen-hui Lou, Wen-chuan Wu

**Affiliations:** ^1^ Department of General Surgery, Zhongshan Hospital, Fudan University, Shanghai, China; ^2^ Department of Pathology, Zhongshan Hospital, Fudan University, Shanghai, China

**Keywords:** pancreatic cancer, αTubulin, overall survival, prognostic biomarker, nomogram

## Abstract

**Background:**

αTubulin, the essential orchestrator of cytoskeletal protein polymers, critical for cell growth and division, motility, signaling development and maintenance of cell shape, plays vital roles in the oncogenesis and progression of various types of cancer, but its role in prognosis of pancreatic cancer patients remains unknown. The aim of this study was to investigate its prognostic value in patients with pancreatic cancer after surgical resection.

**Results:**

αTubulin expression in pancreatic cancer was significantly associated with N classification (*p* = 0.013) and TNM stage (*p* = 0.025). Increased expression of αTubulin in tumoral tissue was associated with decreased overall survival rate (*p* = 0.002). Multivariate Cox regression analysis suggested that αTubulin expression was an independent prognostic indicator for pancreatic cancer except for T and N classification (*p* = 0.002). Using multivariate analysis, αTubulin expression, CA19-9, and N classification were selected to generate the nomogram to predict the 1-year and 3-year overall survival. The c-index of this model was 0.692. The calibration curve for probability of survival showed good agreement between prediction by nomogram and actual observation.

**Methods:**

αTubulin expression was evaluated by tissue microarrays from 124 pancreatic cancer patients and statistically assessed for correlations with the clinical profiles and the prognosis of the patients with pancreatic cancer. The prognostic nomogram was designed to predict 1-year and 3-year overall survival probability.

**Conclusions:**

αTubulin expression might be an independent prognostic factor for pancreatic cancer after surgical resection and could potentially be a high-priority therapeutic target. Incorporating αTubulin expression into CA19-9 and N classification can provide a good prognostic model.

## INTRODUCTION

Accumulating evidence has showed that disorganization and pleomorphism have important roles in cancer, contributing to cancer diagnosis and therapy decision.

Tubulin would be the essential orchestrator of cytoskeletal protein polymers, critical for cell growth and division, motility, signaling development and maintenance of cell shape [1]. Aberrant expression of tubulin has been reported in some human malignancies such as oral cancer [2], breast cancer [3], rectal cancer [4], lung cancer [5], and prostate cancer [6].

Posttranslational modifications of αTubulin can control diverse microtubule functions, such as signaling, trafficking, and cellular tensegrity [7, 8]. Acetylation of αTubulin, a well-known marker of stabilized microtubules, occurs on lysine 40 (K40) by αTubulin acetyltransferase1 [7, 9]. Furthermore, it has been reported that elevated levels of αTubulin acetylation are a sufficient cause of metastatic potential in breast cancer [10]. In addition, prostate cancer cells showed elevated levels of detyrosinated and polyglutamylated αTubulin than normal prostate cells.

Pancreatic cancer is one of the most common malignancies in the world. The 5-year overall survival of the patients with pancreatic cancer is about 7% [11]. Studies have revealed that βIII tubulin is a key player in promoting pancreatic cancer growth and survival, and silencing its expression may be a potential therapeutic strategy to increase the long-term survival of patients with pancreatic cancer [12]. Studies in animal models of primary and metastatic pancreatic cancer models have showed the therapeutic role of the novel vascular-targeting agent ZD6126 that could disrupt the tubulin cytoskeleton of the tumor endothelium [13].

Our previous study has demonstrated that αTubulin is a potential biomarkers for CA19-9 negative pancreatic cancer while the clinical significance of αTubulin and its prognostic value in pancreatic cancer remain obscure. Thus, illumination of the significance of αTubulin expression in pancreatic cancer might provide some additional prognostic information other than the TNM staging system for a further risk stratification and provide guidance for a more precise treatment for pancreatic cancer patients.

In the study, we investigated the expression of αTubulin in pancreatic cancer and its correlation with the clinicopathological characteristics of the patients. Moreover, a predictive nomogram was generated to give the quantitative evaluation for the 1- and 3-year overall survival of the patients with pancreatic cancer after surgery.

## RESULTS

### Characteristics of patients

The detailed characteristics of patients enrolled in this study were listed in Table 1. Overall survival was defined as the interval between surgery and last visit or death. Most patents were male (53.2%) and old (> 60 years, 54.0%). The 1-year and 3-year overall survival rates of this study population were 56.9% and 6.1% respectively.

**Table 1:** Relation between Tubulin1 A expression and clinical characteristics of patients with PDAC

Factor	Patients	Tubulin1A		*P*-value
	No.	Low	High	
Age (years)				0.216
≤ 60	57	16	41	
> 60	67	26	41	
Gender				0.293
Female	58	23	35	
Male	66	19	47	
Localization				0.173
Head/Neck	94	31	63	
Body/Tail	30	11	19	
Neural invasion				0.285
No	67	26	41	
Yes	57	16	41	
Differentiation				0.194
Well	64	19	45	
Poorly	60	23	37	
CA19-9 (U/L)				0.238
< 37	30	7	23	
≥ 37	94	35	59	
T classification				0.477
T1	39	12	27	
T2	65	25	40	
T3	20	5	15	
N classification				
N0	71	31	40	**0.013**
N1	53	11	42	
TNM stage				**0.025**
I	30	11	19	
II	42	20	22	
III	52	11	41	

*P*-value < 0.05 marked in bold font shows statistical significant.

### Innmunohistochemical findings

**Figure 1:**
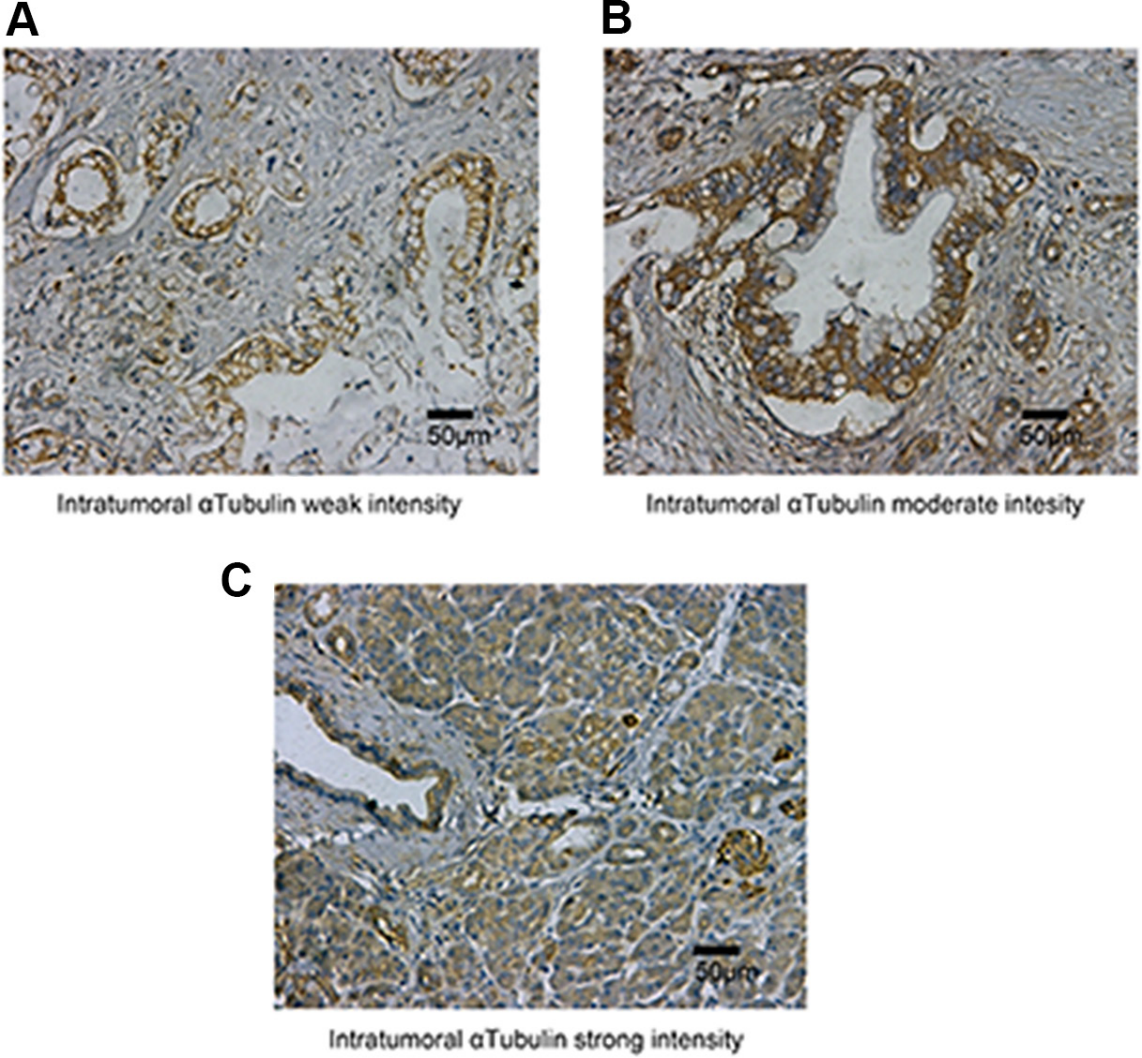
αTubulin expression in tumoral tissue The micrographs showed weak (**A**), moderate (**B**) and strong (**C**) staining of αTubulin in tumoral tissues. Original magnification:×200.

To ascertain the expression of activated αTubulin in pancreatic tumour tissue, we examined the expression of αTubulin in the specimens by IHC staining. The expression of αTubulin was mainly localised in the cell cytoplasm and showed variable staining intensity (Figure 1A–1C).

### Relation between αTubulin expression and clinicopathological features

To evaluate the association of αTubulin expression with tumor biology, comparisons of the clinicopathological features with αTubulin expression were made. As shown in Table 1, αTubulin staining was correlated with N classification (*P* = 0.013) and TNM stage (*p* = 0.025). No association between αTubulin expression and other clinicopathological factors was observed.

### Prognostic significance of αTubulin for pancreatic cancer

**Figure 2:**
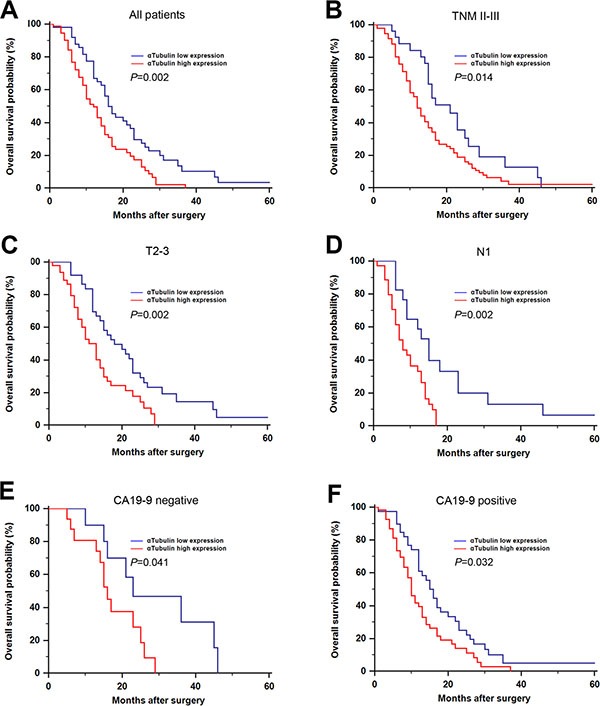
Kaplan–Meier analysis for OS of patients with gastric cancer according to the αSMA expression Kaplan–Meier analysis for OS of patients with gastric cancer according to αSMA expression in all patients (**A**), TNM II-III (**B**), T2-3 (**C**), N1 (**D**), CA19-9 negative (**E**), CA19-9 positive (**F**).

In order to estimate the clinical prognostic significance of αTubulin expression that might influence the overall survival of patients enrolled in this study, Kaplan-Meier survival analysis was performed. As shown in Figure 2, patients with higher expression of αTubulin in tumor tissues were prone to lower OS. Low expression of αTubulin has a survival benefit compared with high expression (Figure 2A, *P* = 0.002). Kaplan-Meier analysis was also applied to compare overall survival according to αTubulin expression in different TNM stage in tumor tissues. Significant difference was found in TNM II-III stage tumor according to αTubulin expression (Figure 2B, *P* = 0.014). Since difference was only found in TNM II-III stage tumors, we gave a further stratified analysis in different T and N classification status. Significant differences were found in T2-3 (Figure 2C, *P* = 0.002) and N1 (Figure 2D, *P* = 0.002) stage tumors. Overall survival for the two subgroups in CA19-9 negative (Figure 2E, *P* = 0.041) and CA19-9 positive (Figure 2F, *P* = 0.032) differed significantly. All these results indicated a vital impact of αTubulin expression on clinical outcome in pancreatic cancer patients, especially for the advanced stage disease. In addition, univariate analyses for overall survival in this study exhibited that high αTubulin expression is a significant negative prognostic predictor for patients with pancreatic cancer (*P* = 0.002, Table 2). Besides, tumor location (*P* = 0.035), N classification (*P* < 0.001), and TNM stage (*P* < 0.001) also significantly affected the survival of patients with pancreatic cancer (Table 2). Furthermore, Cox multivariate regression analyses were performed to derive independent risk estimates related to overall survival. As shown in the Table 2, αTubulin expression (hazard ratio (HR), 1.434; 95% CI, 1.064–1.943; *P* = 0.019), N classification (HR, 2.210; 95% CI, 1.463–3.367; *P* = 0.007), CA19-9 (HR, 1.752; 95% CI, 1.076–2.853; *P* = 0.025) were all recognized as independent prognostic factors.

**Table 2:** Univariate and multivariate analyses of factors associated with survival

	Univariate *P* value	Multivariate	
		HR (95% CI)	*P* value
Age (years): > 60 vs ≤ 60	0.381	NA	NA
Gender: Female vs Male	0.701	NA	NA
Localization: Head/Neck vs Body/Tail	**0.035**	0.643 (0.384–1.076)	0.643
CA19-9 (U/L): ≥ 37 vs < 37	**0.036**	**1.752** (1.076–2.853)	**0.025**
Differentiation: Poorly vs Well	0.259	NA	NA
T classification: T2-3 vs T1	0.307	**NA**	**NA**
T classification: N1 vs N0	**<.001**	**2.210** (1.463–3.367)	**0.007**
TNM stage: II– III vs I	**0.001**		
αTubulin expression: High vs Low	**0.002**	**1.434** (1.064–1.943)	**0.019**

*P*-value < 0.05 marked in bold font shows statistical significant.

### Construction of the nomogram

**Figure 3:**
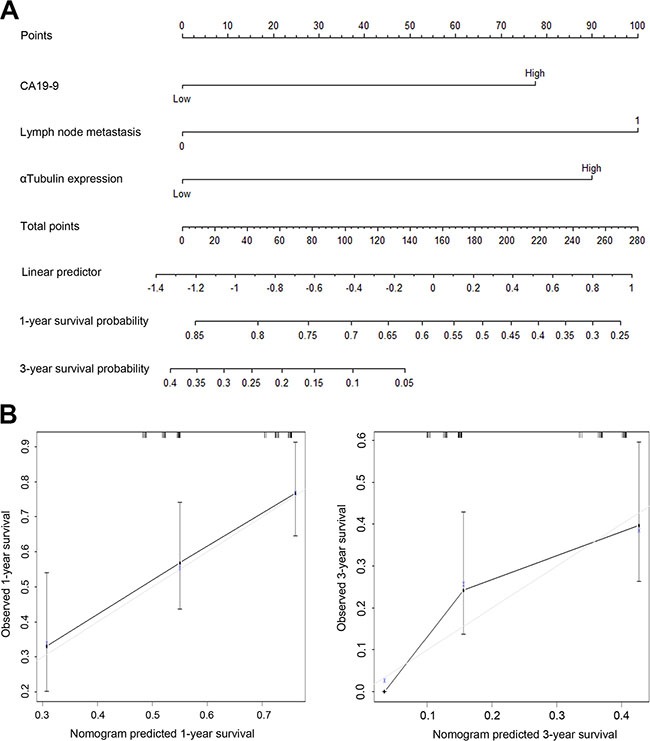
Prognostic nomogram generation for predicting overall survival in patients with gastric cancer (**A**) Nomogram for predicting postoperative 1- year and 3- year survival probabilities after surgery, summing the score of the 3 variables, that is, CA19-9, N classification and αTubulin expression. (**B**) Calibration of the nomogram for 1-year and 3-year overall survival. Bars indicate 95% confidence intervals.

To predict the 1-year and 3-year OS rates of pancreatic cancer, the following three independent variables, including CA19-9, N classification, αTubulin expression, were selected in the nomogram. The sum of the each variable point was plotted on the total point axis, and the estimated median 1-year and 3-year survival rates were obtained by drawing a vertical line from the plotted total point axis straight down to the outcome axis. The c-index of this model was 0.692. Figure 3 showed the calibration graph for the nomogram, in which the probability of 1-year and 3-year survival as predicted by the nomogram is plotted against the corresponding observed survival rates obtained by the Kaplan-Meier method.

## DISCUSSION

In the present study, we investigated αTubulin expression in 124 pancreatic cancer patients, found that high expression in tumor tissue was associated with advanced N classification, advanced TNM stage, and poor clinical outcome. Furthermore, high αTubulin expression was identified as a prognosticator independent of serum CA19-9 level, N classification status. Based on the results, we generated a quantitative nomogram to stratify the patients with different clinical outcomes.

As an important structural component of centrosomes, αTubulin has been reported in various human malignancies. Increasing studies have revealed that αTubulin plays an important role in tumor metastasis [14, 15]. Furthermore, anti-cancer agents target αTubulin causing mitotic arrest and cytotoxicity in different cancers. However, the mechanism of these agents remains unknown. Previous study has showed that phenethy isothiocynate-inducd G2-M cell phase arrest and inhibit the expression of αTubulin in prostatic carcinoma [6]. It has also been reported that phenethyl isothiocyanate and paclitaxel synergistically could enhance apoptosis in breast cancer cell and the effect is associated with elevated level of αTubulin hyperacetylation [16]. In addition, Alhosin has reported that thymoquinone induce a concentration and time dependent degradation of αTubulin in astrocytome. The degradation is related to the up-regulation of the tumor suppressor *P73* with subsequent induction of apoptosis while it has no effect on normal human fibroblast cells. Thus, it is conceivable that aberrant expression of αTubulin in pancreatic cancer tissue could take part in the progression of the primary tumor. In addition, in the present study, we found that high expression of αTubulin would give some additional prognostic information, especially in more advanced tumors, raising the possibility that αTubulin could enhance the progression of tumor. In advanced tumors, more vessels were needed to facilitate nutrition supply and metabolite excretion. Since αTubulin is associated with lymph node stage, we could give a reasonable explanation for our result that high αTubulin expression in pancreatic cancer was associated with advanced tumor stage.

CA19-9 is a tumor-associated antigen, initially identified in the sera of patients with pancreatic and colon malignancies. Currently, CA19-9 is the most important biomarker for the diagnosis, prognosis and management of PDAC [17–24], whose sensitivity and specificity for pancreatic cancer both are approximately 80% [25], but it is not a sensitive marker to detect PDAC in the early stage. Furthermore, CA19-9 reacts with the sialylated Lewisa blood group antigen present in the glycoprotein serum fraction [26]. However, approximately 5% to 10% of the general population has the Lewis a-b- phenotype, which means that they are unable to synthesize the CA19-9 antigen and will not have elevated levels secondary to pancreatic cancer [27]. This raised the hypothesis that tumors of the two subtypes could take place under different biological circumstances, although they both are cancers in the pancreas.

We unraveled the prognostic value of αTubulin expression in pancreatic cancer especially in more advanced tumors. However, there are several limitations of this study. First, this study is limited by the retrospective nature of the analysis and the selection biases cannot be totally eliminated. Second, there is not including the data of disease free survival in this study. There are many factors, such as the follow-up examinations and the postoperative treatment, might influence the disease free survival. And the disease free survival data should be collected in the future researches. Third, the results were mainly based on the expression of αTubulin by means of IHC staining, the exact mechanisms would be investigated in our future work. Finally, the number of patients included in this study is relatively small. Large prospective randomized controlled clinical studies are needed to identify the prognostic value of αTubulin expression in the patients with pancreatic cancer.

In conclusion, we have identified increased expression of αTubulin in pancreatic cancer as an independent unfavorable prognostic factor, which could be integrated with depth of tumor invasion, N classification status, and distant metastasis status to generate a nomogram to give a better risk stratification for pancreatic cancer patients with different prognosis, especially in more advanced stages.

## MATERIALS AND METHODS

### Patients and specimens

Between January 2005 and December 2009, a total of 124 patients underwent surgical resection of pancreatic cancer were collected in the Department of General Surgery of Zhongshan Hospital of Fudan University (Shanghai, China). Specimens were reassessed by two pathologists independently. A retrospective review of clinical data was performed, and the clinicopathological features (patient's age, gender, tumor localization, degree of tumor differentiation, neural invasion, and TNM stage) and the oncological results (overall survival rate) were analyzed. The follow-up was conducted until the November 31, 2014 or until death. No patients had been lost to follow-up. Ethical approval was granted by the Clinical Research Ethics Committee of Zhongshan Hospital of Fudan University (Shanghai, China). Signed informed consent was obtained from all patients for the acquisition and use of anonymized clinical data.

### Tissue microarray and immunohistochemistry

Formalin-fixed and paraffin-embedded surgical specimens were used for tissue microarray construction and subsequent IHC study. The IHC were performed as described previously [28]. The primary antibody against αTubulin (Abcam, Cambridge, MA, USA) was used for IHC analysis. All the cases were stained at once. The sections were scanned at ×200 magnification. Image-Pro Plus version 6.0 software (Media Cybernetics Inc., Bethesda, MD) was used to measure the density of the positive staining. The intensity of immunohistochemical staining of αTubulin was scored by two pathologists using the semi-quantitative immunoreactivity scoring (IRS) system as described previously [29]. Immunoreactivity score was derived by multiplying the intensity of immunohistochemical staining and the percentage of immunoreactive cells ranged from 0 to 12, and we defined 6 as the cutoff value for high and low expression according to the ‘minimum P-value method’ on the basis of its relation with OS. The negative control staining was treated equally with the primary antibody omitted.

### Statistics

Statistical analyses were performed using SPSS Software (version 19.0; SPSS Inc., Chicago, IL, USA) and R 3.2.0 software (https://www.r-project.org/). The statistical significance of categorical data was evaluated using χ^2^ test or *t* test as appropriate. Cumulative survival time was calculated by Kaplan-Meier method and analyzed by log-rank test. The Cox proportional hazards regression model was used to perform univariate and multivariate analyses in order to determine the independent prognostic factors, and the Cox model was the basis for the nomogram. We also performed calibration using a calibration curve, a graphic representation of the relationship between the observed outcome frequencies and the predicted probabilities. All data were analyzed using two-tail test and *P* < 0.05 was considered statistically significant.
